# Differential regulation of MAP kinase cascade in human colorectal tumorigenesis

**DOI:** 10.1038/sj.bjc.6690817

**Published:** 1999-12

**Authors:** K-S Park, N-G Kim, J J Kim, H Kim, Y H Ahn, K-Y Choi

**Affiliations:** Department of Biochemistry and Molecular Biology, Institute of Genetic Sciences andDepartment of Pathology, Yonsei University College of Medicine, Seoul 120-752, Korea

**Keywords:** colorectal cancer, extracellular signal regulated kinase, Ras, MEK, Raf-1

## Abstract

Hyper-activation of mitogen-activated protein kinase (MAPK) has recently been reported in several human cancers and activation of MAPK in those cancers may be associated with carcinogenesis through aberrant cell proliferation. To understand the roles of the MAPK pathway in colorectal tumorigenesis, we examined the status of extracellular signal-regulated protein kinases (ERK1/2) in 21 colorectal tumour specimens and compared it with that of paired normals. The specific MAPK activities were two- to tenfold lower in 71% (15 out of 21 cases) of colorectal tumours compared to those in paired normals. The individual MAPK kinase (MEK) correlated with MAPK activities (*P* = 0.006). Reduction of the MAPK and MEK activities in colorectal tumours was also observed in adenomas. These results suggested that down-regulation of the MAPK cascade may be caused by early genetic event(s) and that it may be related to the loss of normal growth control. Although MAPK activities were down-regulated both in adenomas and carcinomas, activities of the MAPKs in carcinomas were higher than those of paired adenomas. These results suggested that MAPK activities may be increased in the adenoma-to-carcinoma sequence and that it may play a role in the tumour progression. Observation of the differential regulation of MAPK activities in colorectal tumorigeneis suggested roles for the MAPK pathway in both positive and negative controls of cell growth. © 1999 Cancer Research Campaign


*British Journal of Cancer* (1999) **81**(7), 1116–1121
© 1999 Cancer Research Campaign
Article no. bjoc.1999.0817

### Differential regulation of MAP kinase cascade in human colorectal tumorigenesis

**K-S Park[1,2], N-G Kim[3], JJ Kim[3], H Kim[3], YH Ahn[1,2] and K-Y Choi[1,2]**

[1]Department of Biochemistry and Molecular Biology, [2]Institute of Genetic Sciences and [3]Department of Pathology, Yonsei University College of Medicine, Seoul 120-752, Korea

**Summary** Hyper-activation of mitogen-activated protein kinase (MAPK) has recently been reported in several human cancers and activation of MAPK in those cancers may be associated with carcinogenesis through aberrant cell proliferation. To understand the roles of the MAPK pathway in colorectal tumorigenesis, we examined the status of extracellular signal-regulated protein kinases (ERK1/2) in 21 colorectal tumour specimens and compared it with that of paired normals. The specific MAPK activities were two- to tenfold lower in 71% (15 out of 21 cases) of colorectal tumours compared to those in paired normals. The individual MAPK kinase (MEK) correlated with MAPK activities ($P = 0.006$). Reduction of the MAPK and MEK activities in colorectal tumours was also observed in adenomas. These results suggested that down-regulation of the MAPK cascade may be caused by early genetic event(s) and that it may be related to the loss of normal growth control. Although MAPK activities were down-regulated both in adenomas and carcinomas, activities of the MAPKs in carcinomas were higher than those of paired adenomas. These results suggested that MAPK activities may be increased in the adenoma-to-carcinoma sequence and that it may play a role in the tumour progression. Observation of the differential regulation of MAPK activities in colorectal tumorigeneis suggested roles for the MAPK pathway in both positive and negative controls of cell growth. © 1999 Cancer Research Campaign

**Keywords:** colorectal cancer; extracellular signal regulated kinase; Ras; MEK; Raf-1

Mitogen-activated protein kinases (MAPKs), also known as extracellular signal-regulated kinases (ERK1/2), are essential components of the MAPK signalling pathway. MAPKs are activated in response to various stimuli, including growth factors, cytokines, hormones and neurotransmitters, and their activities are a determining factor for cell proliferation and differentiation (Blenis, 1993; Stevenson et al, 1994; Marshall, 1995). The activation of ERK1/2 is the result of the sequential activation of a series of upstream kinases, including Raf-1 and MEK (Cobb and Goldsmith, 1995). Activation of ERK1/2 requires dual phosporylation on threonine and tyrosine residues of the specific peptide motif, Thr–Glu–Tyr (Zheng and Kuan, 1993). The phosphorylation of ERK1/2 is a reversible process which is mediated by dual specific threonine–tyrosine phosphatases like MAPK phosphatase 3 (MKP-3) (Groom et al, 1996). Activated MAPKs transmit their signals into the nucleus for activation of target transcription factors such as c-Jun, c-Fos (Lange-Carter et al, 1993) and Elk-1 (Marais et al, 1993). Accurate regulation of MAPK signal transduction is essential for normal cell growth control, and abnormalities in the components of the MAPK pathway are often observed in various human cancers (Owen et al, 1984; Bos, 1989; Slamon et al, 1989; Fearon and Vogelstein, 1990; Janes et al, 1994). Although ERK1/2 are key components in the MAPK pathway, the relationship between abnormalities of ERK1/2 and human cancers has not been well characterized. Hyper-expression and activation of ERK1/2 MAPKs have recently been reported in several human cancers, including breast carcinomas, leukaemia and renal cell carcinomas (Oka et al, 1995; Sivaraman et al, 1997; Towatari et al, 1997). However, it is not known whether these are generalized characteristics for every type of cancer.

Tumorigenesis of the colon is a multistep process including various genetic alterations of oncogenes (K-*ras*, *src* and *myc*) and tumour suppressor genes (*APC*, *p53* and *DCC* etc.) (Fearon and Vogelstein, 1990; Simon et al, 1994; Kinzler and Vogelstein, 1996). The K-*ras* gene mutations, which appear in 50% of intermediate adenomas (greater than 1 cm), are one of the major causes of colon carcinogenesis, suggesting that MAPK activation is required for tumour progression (Kinzler and Vogelstein, 1996). Therefore, it is possible that hyper-activation of ERK1/2 MAPKs may exist in colorectal tumours.

In this study, we examined the status of ERK and MEK kinases in colorectal specimens to elucidate the relationship between colorectal carcinogenesis and the MAPK signalling pathway. Here, we report unusual differential regulation of the MAPK pathway during colorectal tumorigenesis, suggesting possible roles of the ERK pathway in both positive and negative growth control.

*Received 9 February 1999*
*Revised 6 May 1999*
*Accepted 10 May 1999*

*Correspondence to:* K-Y Choi, Department of Biochemistry and Molecular Biology, Yonsei University College of Medicine, 134 Shinchon-Dong Seodemun-Gu, Seoul 120-752, Korea

### MATERIALS AND METHODS

### Human tissues and patient information

A total of 21 colorectal carcinomas and seven adenomas (several of them had different paired adenomas) and adjacent paired

**Table 1** Inhibition of ERK and MEK activities in human colorectal tumour

| Case | Tumour | | Relative MAPK inhibition | | Relative MAPK expression[c] | MEK inhibition[d] |
|---|---|---|---|---|---|---|
| | Grade (differentiation) | Stage | In vitro kinase assay[a] | Phosphorylation[b] | | |
| 1 | Well | 3 ($T_3N_0M_0$) | 10.42 | 4.8 | 1.29 | ND |
| 2 | Well | 2 ($T_3N_0M_0$) | 6.37 | 3.2 | 1.0 | + |
| 3 | Moderate | 2 ($T_2N_0M_0$) | 6.19 | 5.0 | 1.1 | + |
| 4 | Moderate | 2 ($T_3N_0M_0$) | 6.19 | 4.0 | 0.93 | + |
| 5 | Moderate | 2 ($T_3N_0M_0$) | 5.27 | 3.0 | 0.9 | + |
| 6 | Moderate | 2 ($T_3N_0M_0$) | 5.05 | 2.5 | 1.08 | + |
| 7 | Moderate | 3 ($T_3N_0M_0$) | 5.05 | 5.0 | 0.87 | + |
| 8 | Moderate | 3 ($T_3N_1M_0$) | 4.8 | 6.4 | ND | + |
| 9 | Moderate | 2 ($T_3N_0M_0$) | 3.8 | ND | 1.0 | + |
| 10 | Moderate | 2 ($T_3N_0M_0$) | 3.5 | 6.8 | 1.3 | + |
| 11 | Well | 3 ($T_3N_2M_0$) | 3.16 | 3.1 | ND | + |
| 12 | Poor | 3 ($T_3N_1M_0$) | 3.16 | 3.0 | 1.07 | + |
| 13 | Moderate | 2 ($T_3N_0M_0$) | 3.0 | 3.3 | 1.5 | + |
| 14 | Moderate | 2 ($T_3N_0M_0$) | 2.4 | 2.0 | 1.0 | + |
| 15 | Moderate | 2 ($T_3N_0M_0$) | 2.8 | 1.0 | 1.5 | – |
| 16 | Poor | 2 ($T_2N_0M_0$) | 1.53 | 1.5 | 1.5 | – |
| 17 | Moderate | 2 ($T_3N_0M_0$) | 1.5 | 1.5 | 1.14 | – |
| 18 | Moderate | 3 ($T_4N_1M_0$) | 1.5 | 0.5 | 1.32 | + |
| 19 | Moderate | 3 ($T_3N_2M_0$) | 1.15 | 1.5 | 1.01 | – |
| 20 | Moderate | 2 ($T_3N_0M_0$) | 1.01 | 1.0 | 0.67 | – |
| 21 | Moderate | 2 ($T_2N_0M_0$) | 1 | 2.0 | 1.19 | – |

[a]Relative MAPK inhibitions were obtained by N/C ratio of MAPK activity obtained by quantitation of phospho Elk-1 bands produced by in vitro kinase assay (see Materials and Methods). Representative cases of the assay are shown in Figure 1. [b]Relative MAPK inhbitions were obtained by N/C ratio of phospho-ERK1/2 band intensities produced by Western blot analysis. Representative cases of the assay are shown in Figure 2A. [c]Relative MAPK expressions were obtained by N/C ratio of MAPK band intensities produced by Western blot analysis. Representative cases of the assay are shown in Figure 2B. [d]Relative MEK inhibition values were obtained by N/C ratio of specific MEK activities (phospho-MEK/MEK band intensities) produced by Western blot analysis. Representative cases of the assay are shown in Figure 4. The cases which show N/T ratio higher than 2 were recorded as positive. 'ND' represent not determined data.

normal mucosa tissues from 21 patients, as well as two adenoma and paired normal mucosa from a familial adenomatous polyposis (FAP) patient, were included in this study. This work was performed after approval by the Human Investigation Committee of the Yonsei University College of Medicine. All tissue donors agreed to provide their samples for this study. The tissues from surgically resected specimens were fractionated separately for biochemical analysis and stored at –80°C. Five breast tissue specimens of each cancer and paired normal were also prepared in a similar way. Information from the Yonsei University Tumor Registry and chart review was obtained to determine the demographics and tumour sites of the patients: 14 were at stage 2 and seven were at stage 3 (Table 1). The six carcinomas located proximal to the splenic flexure were classified as right-sided, and the 15 distal to the splenic flexure as left-sided. The genetic defects reported in colorectal cancers were also observed in our cases with similar frequencies. Microsatellite instability of tumours was observed in six of 21 cases (29%), and p53 mutations (analysed by SSCP of exon 5–9) were observed in 13 of 21 cases (62%). Furthermore, 52% (11 of 21 cases) of K-*ras* had a mutation in codon 12 or 13.

### Extract preparation

Frozen tissue samples were pulverized in liquid nitrogen and the resultant powder was dissolved in ice-cold lysis buffer (70 mM β-glycerophosphate pH 7.2, 1 mM each meta- and ortho-vanadate, 2 mM magnesium chloride ($MgCl_2$), 1 mM EGTA, 1 mM dithiothreitol (DTT), 0.5% Triton X-100, 0.2 mM phenylmethylsulphonyl fluoride (PMSF) and 5 μg $ml^{-1}$ each of pepstatin A, chymostatin, leupeptin and peptin) and kept on ice for 20 min. The samples were sonicated for 30 s and unbroken cellular debris was removed by centrifugation at 23 000 ***g*** for 10 min. The supernatant was further cleared by a subsequent centrifugation. Samples were immediately aliquoted and frozen at – 80°C. Protein concentrations were determined with a Bio-Rad protein assay method (Bio-Rad Laboratories, Richmond, CA, USA).

### In vitro MAPK assays

MAPK assays were performed by a non-radioactive method developed by New England Biolabs Inc. (Beverly, MA, USA) as recommended by the manufacturer. MAPKs were immunoprecipitated from 300 μg of tissue extract with phospho-specific ERK1/2 MAPK (Thr202/Tyr204) monoclonal antibody, and kinase assays were performed in the presence of ATP and a GST-fused physiological substrate, Elk-1. Detection of the phospho-Elk-1 protein was performed by Western blot analysis using phospho-specific Elk-1 (Ser383) antibody. The phospho Elk-1 antibody specifically recognizes Elk-1 protein with phosphorylation at Ser383.

### Western blot analysis

The lysates containing 40–100 μg of protein were subjected to 8%, 10%, or 12% sodium dodecyl sulphate polyacrylamide gel electrophoresis (SDS-PAGE; acrylamide:bis-acrylamide at a ratio of 29:1) and proteins were transferred onto a Protran Nitrocellulose Membrane (Schleicher and Schuell Corporation, Dassel,

© 1999 Cancer Research Campaign

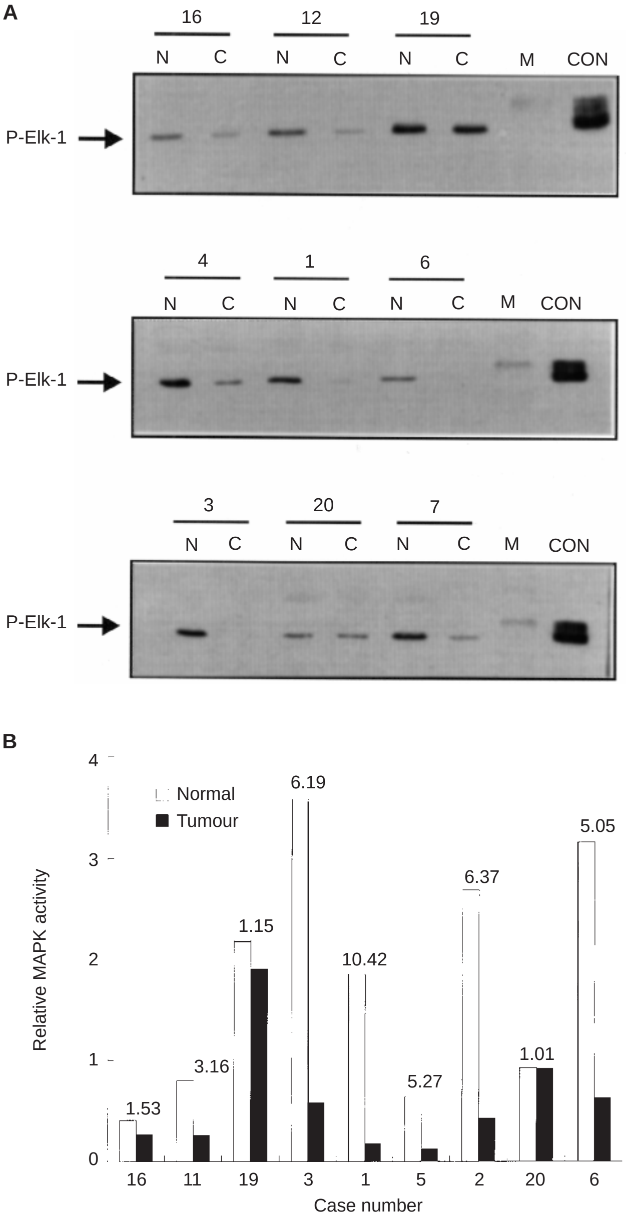

**Figure 1** Relative MAPK activities in representative colorectal tumours and paired normals. (**A**) MAPK activity was determined by the ability of phospho-specific MAPK (Thr202/Tyr204) antibody-immunoprecipitates to phosphorylate Elk-1 as described in Materials and Methods. The phospho-Elk-1 was detected by Western blot analysis using phospho-specific Elk-1 (Ser383) antibody. Numbers above the figure represent case numbers. N represents normal tissue and C represents carcinoma tissue. The M represents biotinylated protein-sized marker, and Con represents Elk-1 protein phosphorylated by activated P-ERK1/2. (**B**) Quantitative comparison of MAPK activities. MAPK activities were quantitated by densitometric scanning of ECL film as described in Materials and Methods. Numbers above the bars represent fold-inhibition calculated by N/C ratio

Germany). Blots were blocked in 20 mM Tris–Cl, pH 7.5, 150 mM sodium chloride (NaCl), 0.05% Tween-20 (TBST) containing 5% non-fat Carnation milk. Immunoblots were then washed with TBST and incubated 2 h at room temperature or overnight at 4°C with TBST milk containing appropriate amounts of primary antibodies. The blots were then washed and incubated with TBST milk containing appropriate secondary antibody for 1–2 h. After washing, the blots were developed with Amersham ECL kit (Amersham International, Buckinghamshire, UK). Quantitation of Western bands were made by densitometric scanning of the films by using a Bio-Rad imager system (Model GS-525) with analysing software (Molecular Analyst Software version 1.5, BioRad). MEK inhibition was considered as positive when the specific MEK activity of a tumour was lower (> twofold) than that of a paired normal (Table 1).

### Antibodies

The following antibodies were purchased and used for Western blot analysis and immunoprecipitation. Anti-MAPK rabbit polyclonal antibody was purchased from Stratagene (La Jolla, CA, USA). Phospho-specific MAPK antibody (raised against synthetic phospho-tyrosine peptide corresponding to residues 196 to 209 [DHTGFL TEY(P) VATRWC] of human ERK2] was purchased from New England Biolabs Inc. Phospho-specific MEK antibody (produced by immunizing rabbits with synthetic phospho-Ser217/221 peptide) was also purchased from New England Biolabs, Inc. Anti-MEK antibody was purchased from Transduction Laboratories (Lexington, KY, USA). Horseradish peroxidase-conjugated secondary antibodies were purchased from New England Biolab Inc. or Transduction Laboratories.

### Statistical analysis

Linear correlation between two variables of MAPK inhibition (in vitro kinase assay versus phospho-ERK1/2) was determined by calculating Pearson's correlation coefficient. The Wilcoxon's rank sum tests were performed to determine the relationships between MAPK inhibition and phospho-MEK. *P*-values less than 0.05 were considered to be statistically significant. Data were analysed with SPSS statistical software package (SPSS Inc., Chicago, IL, USA).

### RESULTS

### Inhibition of MAPK activity in colorectal tumour

To elucidate the relationship between MAPK and colorectal tumours, we measured ERK1/2 MAPK activities in 21 human colorectal tumour tissues and paired normals. The ERK1/2 MAPK assays were performed using Elk-1 as a substrate (Marais et al, 1993). In addition, MAPK activity was also analysed alternatively by measuring the level of phospho-ERK1/2 (Oka et al, 1995; Sivaraman et al, 1997). To our surprise, MAPK activities were significantly lower in 71% of the tumours (15 of 21 cases) compared to those in paired normals (Table 1). Nine representative cases are shown in Figure 1. The degree of inhibition (N/C ratio > 2) varied from tumour to tumour, with a two- to tenfold variation (Table 1). The phospho-ERK1/2 MAPKs were also lower in 70% of tumours (14 of 20 cases), and the levels were linearly correlated with MAPK activities ($P$ = 0.002; Table 1). Five representative cases are shown in Figure 2A. However, the levels of ERK1/2 proteins were similar in all cases including both tumours and normals (Figure 2B and Table 1). The inhibition of ERK1/2 activities did not significantly correlate with the location, grade, stage and other pathological states of the tumours (see Materials and Methods).

 © 1999 Cancer Research Campaign

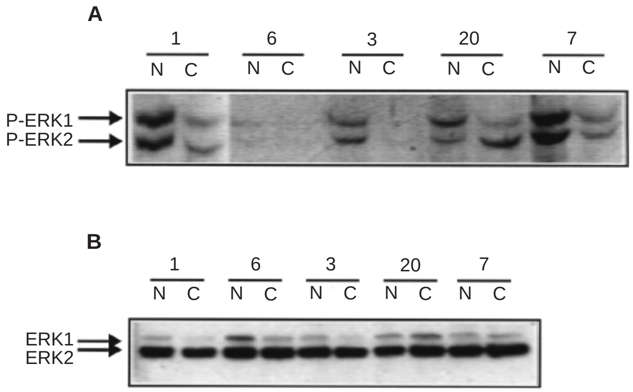

**Figure 2** Levels of MAPKs in the representative colorectal tumours and paired normals. (**A**) The phospho-ERK proteins were detected by Western blot analysis by using phospho-specific MAPK (P-ERK1/2) antibody. (**B**) The ERK1/2 proteins were detected by Western blot analysis by using ERK1/2 polyclonal antibody. N and C also represent normal and carcinoma respectively

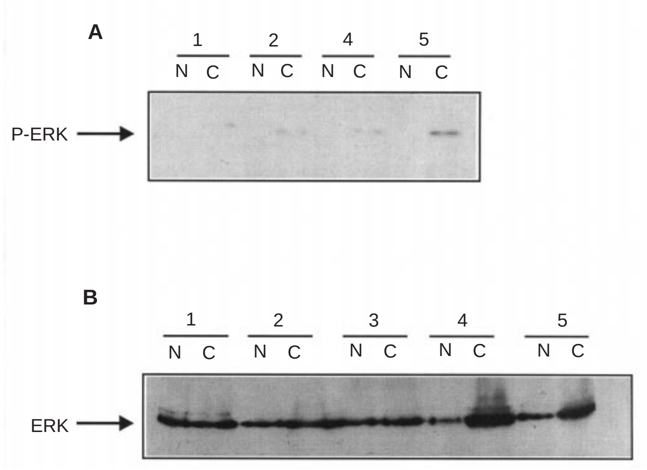

**Figure 3** MAPK activities and the expression of MAPKs in breast tumours and paired normals. (**A**) MAPK activity was detected by Western blot analysis by using phospho-specific ERK1/2 antibody. (**B**) The ERK proteins were detected by Western blot analysis by using ERK1/2 polyclonal antibody. Numbers above the lanes represent case numbers. C represents tissue from breast carcinoma patients, and N represents normal tissue from the patient

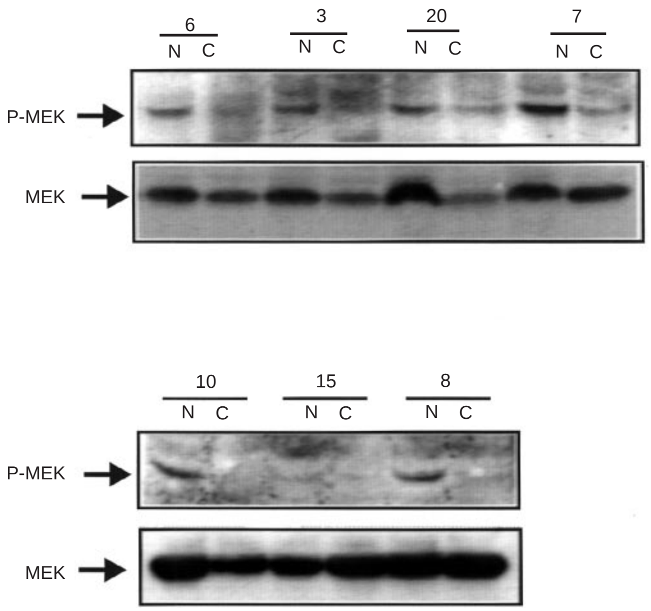

**Figure 4** Inhibition of MEK activity in representative colorectal tumours and paired normals. MEK activity was monitored by Western blot analysis using phospho-specific MEK polyclonal antibody. MEK protein levels were detected by Western blot analysis using MEK polyclonal antibody. Numbers represent case numbers. N and C also represent normal and carcinoma respectively

### Activation MAPK activity in breast carcinoma

Since the low MAPK activity in the colorectal tumours was unexpected, we also checked ERK1/2 activities in five breast carcinomas and five normal tissues from the same patient by checking phospho-ERK1/2 levels (Sivaraman, 1997). In contrast to the cases of colorectal tumour, ERK activities were increased in all four breast carcinomas compared to those of paired normals (Figure 3A). In addition, two of five cases (nos 4 and 5) of breast carcinomas showed overexpression of ERK (Figure 3B).

### Inhibition of MEK in colorectal tumour

To determine a source for the inhibition of ERK1/2 activities, we measured activities of an upstream kinase, MEK, by measuring the levels of phospho-MEK. The phospho-MEK protein levels were also lower in 70% (14 out of 20 cases) of the colorectal tumours (Table 1). Seven representative cases of these are shown in Figure 4. The MEK protein levels were similar or slightly higher in normals, except case no. 20, which showed a two- to threefold higher protein level in normal (Figure 4). Inhibition of specific MEK activities was correlated with the level of MAPK activity ($P = 0.0006$) (Table 1).

### Inhibition and activation of ERK1/2 and MEK activities during colorectal tumorigenesis

Due to the unexpected depression of ERK1/2 activities in colorectal carcinomas, we were interested in ERK1/2 activities in the adenomas and measured the MAPK activities in seven adenomas by measuring phospho-ERK1/2 protein levels. The size of adenomas varied from 0.4 to 2.8 cm (Figure 5). Several cases had multiple-sized adenomas (Figure 5; nos 22, 13 and 2). In adenomas, ERK1/2 activities were also significantly down-regulated in six of seven cases (nos 10, 15, 8, 11, 13 and 2) and the ERK1/2 activities in adenomas were even lower than those in carcinomas, except in case nos 8 and 13 (Figure 5). The ERK1/2 activities in case no. 22, a patient with FAP, were significantly lower even in normal samples. Interestingly, the ERK activities in carcinomas were higher than paired adenomas in four (nos 10, 15, 11 and 2) out of six cases which showed down-regulation of enzyme activities in adenomas. The variations of phospho-ERK1/2 in different steps of tumorigenesis mostly correlated well with those of phospho-MEK activities, and several available cases including the normal and 0.8 cm adenoma in the FAP are shown in Figure 5. Although phospho-ERK and phospho-MEK levels were variable, ERK and MEK levels were mostly equivalent in the different colorectal specimens.

© 1999 Cancer Research Campaign

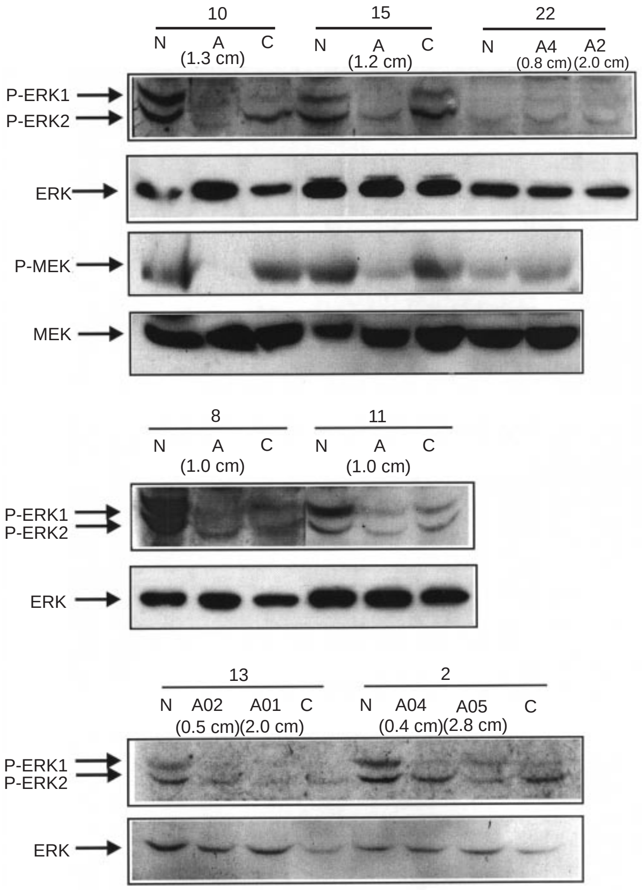

**Figure 5** Differential MAPK and MEK activities in colorectal tumorigenesis. The ERK1/2 and MEK activities in representative adenomas, paired normals and carcinomas were detected by Western blot analysis by using phospho-specific ERK1/2 and MEK antibodies. The ERK and MEK proteins were detected by Western blot analysis by using ERK1/2 or MEK antibody. Numbers in brackets represent the size of adenomas. Numbers above the cases represent case numbers. Each N, A and C denotes normal, adenoma and carcinoma respectively

### DISCUSSION

The *ras* gene product, Ras, functions upstream of Raf-1 in the MAPK pathway and its status is an important factor in the activation of ERK1/2 (Blenis, 1993; Marais et al, 1993). Point mutations of the *ras* oncogene cause the Ras protein to remain in an active state, continuously sending signals downstream (Vogel et al, 1988). Like other cancers, overexpression and mutations of the *ras* gene are frequently observed in colorectal tumours (Spandidos, 1984; Vogelstein et al, 1988; Ahnen et al, 1998). Therefore, we questioned the activation status of ERK1/2 MAPKs in colorectal cancer. Interestingly, ERK1/2 activities were significantly lower in 71% of tumours compared to those in paired normal mucosa tissues. However, ERK1/2 protein levels were similar in all cases, including both cancers and paired normals. Therefore, the differential ERK1/2 activities may be due to the modification of protein, phosphorylation for example, rather than differential gene expression or protein stability. ERK activities in colorectal cells were mostly proportional to the protein levels of phospho-ERKs (Table 1). Low ERK activities in our colorectal tumours were surprising considering that ERK activities are increased in breast carcinomas, renal cell carcinomas and leukaemia (Oka et al, 1995; Sivaraman et al, 1997; Towatari et al, 1997). Therefore, we also checked the status of ERKs in breast carcinoma tissues where increased ERK activities were observed (Sivaraman et al, 1997). The levels of phospho-ERK were significantly higher in all four cases of breast carcinoma compared with those in paired normals. Similar to the previous study, the ERK protein levels in breast carcinomas were also elevated in several cases, which were not observed in any of our 21 colorectal carcinomas. Therefore, depressed activities of ERKs in colorectal cancer are not a common feature of all cancers.

To investigate the source of ERK inactivation in colorectal tumours, we checked the activities in the upstream signalling components MEK by monitoring the status of protein modifications. The MEK activities measured by phospho-MEK immunoblot were mostly lower in colorectal tumours, as were the cases of MAPK activities. Furthermore, patterns of MEK activation in adenoma-to-carcinoma sequences were also in agreement with those of ERK activation. Therefore, a signal for ERK inhibition may come from upstream MEK. The inhibition of ERK activities in a colorectal tumour is highly analogous to that of PKC. The activities of protein kinase C (PKC) were also reduced in human colon carcinoma when those were compared with paired normal mucosa (Guillem et al, 1987; Kopp et al, 1991). In addition, the decrease in PKC activity in adenomas was also similar to the case of MAPK activity, in that it was related to the occurrence of early premalignant stages of intestinal mucosa (Kopp et al, 1991). Although limited by the number of cases, down-regulation of ERK activities in adenocarcinoma and correlation of the ERK activities with PKC activities was observed in a previous study (Attar et al, 1996). In addition, reduced MAPK activity in human gastric adenocarcinoma was also reported (Atten et al, 1995).

Physiological roles of the high ERK activities in normal colorectal mucosa were not understood. However, our results of the reduction of ERK and MEK activities in early adenomas suggested that ERK activities in normal colonic epithelial cells may be involved in normal cellular growth control (Atten et al, 1995), and that the early genetic events in tumorigenesis could result in the lack of growth control, which may be related to the reduction of ERK activities. A role of ERK activation in growth inhibition rather than growth stimulation were recently reported (Pumiglia and Decker, 1997; Sewing et al, 1997). Down-regulation of the ERK pathway in early adenoma may result in the activation of a circumventing mechanism for aberrant cell growth like *c-MYC* overexpression (Alexander et al, 1989; Marcus et al, 1992; He et al, 1998), and that together with secondary genetic events like *ras* mutation may promote the progression to malignancy. In colorectal tumorigenesis, most K-*ras* mutations occurred in the mid-adenoma (larger than 1 cm) stages of tumorigenesis and a *ras* mutation may still be required in the progression to colorectal carcinoma (reviewed in Fearon and Vogelstein, 1990; Kinzler and Vogelstein, 1996). Therefore, it is possible that an increase of ERK and MEK activities in adenoma-to-carcinoma sequence of colorectal tumorigenesis may be caused by a *ras* mutation. A role of *ras* mutations in tumour progression, not in the initiation, was also suggested (Kinzler and Vogelstein, 1996). Dramatic down-regulation of ERK1/2 and MEK activities in the early stages of adenoma suggest the possible application of this phenomenon for early diagnosis of colorectal tumour.

 © 1999 Cancer Research Campaign

### ACKNOWLEDGEMENTS

We wish to thank Robert Ross for his editorial assistance in English. We also thank Sun-Hong Kim and Jonggun Lim for their help with technical assistance and statistical analysis respectively. We also thank Kyung-Sup Kim for his helpful discussion. This work was supported by a grant of "96–98" Good Health R & D project (Grant # HMP-96-M-2-1019 to KYC and HK), Ministry of Health and Welfare, R.O.K.

### REFERENCES

Ahnen DJ, Feiglm P, Quan G, Fenoglio-Preiser C, Lovato LC, Bunn Jr PA, Stemmerman G, Wells JD, Macdonald JS and Meyskens Jr FL (1998) Ki-*ras* mutation and p53 overexpression predict the clinical behavior of colorectal cancer: a Southwest Oncology Group study. *Cancer Res* **58**: 1149–1158

Alexander WS, Adams JM and Cory S (1989) Oncogene corporation in lymphocyte transformation: malignant conversion of E mu-myc transgenic pre-B cells in vitro is enhanced by v-H-ras or v-raf but not v-abl. *Mol Cell Biol* **9**: 67–73

Attar BM, Atten MJ and Holian O (1996) MAPK activity is down-regulated in human colon adenocarcinoma: correlation with PKC activity. *Anticancer Res* **16**: 395–400

Atten MJ, Attar BM and Holian O (1995) Decreased MAP kinase activity in human gastric adenocarcinoma. *Biochem Biophys Res Commun* **212**: 1001–1006

Blenis J (1993) Signal transduction via the MAP kinases: proceed at your own RSK. *Proc Natl Acad Sci USA* **90**: 5889–5892

Bos JL (1989) Ras oncogenes in human cancer. *Cancer Res* **49**: 4682–4689

Cobb MH and Goldsmith EJ (1995) How MAP kinases are regulated. *J Biol Chem* **270**: 14843–14846

Dent P, Jelinek T, Morrison DK, Weber MJ and Sturgill TW (1995) Reversal of Raf-1 activation by purified and membrane-associated protein phosphatase. *Science* **268**: 1902–1905

Fearon ER and Vogelstein BA (1990) Genetic model for colorectal tumorigenesis. *Cell* **61**: 759–767

Groom LA, Sneddon AA, Alessi DR, Dowd S and Keyse SM (1996) Differential regulation of the MAP, SAP and RK/p38 kinases by Pyst1, a novel cytosolic dual-specificity phosphatase. *EMBO J* **15**: 3621–3632

Guillem JG, O'Brian CA, Fitzer CJ, Frode KA, LoGerfo P, Treat M and Weinstein IB (1987) Altered levels of protein kinase C and $Ca^{2+}$-dependent protein kinases in human colon carcinomas. *Cancer Res* **47**: 2036–2039

He T-C, Sparks BAB, Rago C, Hermeking H, Zawel L, da Costa LT, Morin PJ, Vogelstein B and Kinzler KW (1998) Identification of c-MYC as a target of the APC pathway. *Science* **281**: 1509–1512

Janes PW, Daly RJ, deFazio A and Sutherland RL (1994) Activation of the Ras signaling pathway in human breast cancer cells overexpressing *erb*B-2. *Oncogene* **9**: 3601–3608

Kinzler KW and Vogelstein B (1996) Lessons from hereditary colorectal cancer. *Cell* **87**: 159–170

Kopp R, Noelke B, Sauter G, Schildberg FW, Paumgartner G and Pfeiffer A (1991) Altered protein kinase C activity in biopsies of human colonic adenomas and carcinomas. *Cancer Res* **51**: 205–210

Lange-Carter CA, Pleiman CM, Gardner AM, Blumer KJ and Johnson GL (1993) A divergence in the MAP kinase regulatory network defined by MEK kinase and Raf. *Science* **260**: 315–319

Magnuson NS, Beck T, Vahidi H, Hahn H, Smola U and Rapp UR (1994) The Raf-1 serine/threonine protein kinase. *Semin Cancer Biol* **5**: 247–253

Marais R, Wynne J and Treisman R (1993) The SRF accessory protein Elk-1 contains a growth factor-regulated transcriptional activation domain. *Cell* **73**: 381–393

Marais R, Light Y, Mason C, Paterson H, Olson MF and Marshall CJ (1998) Requirement of Ras-GTP-Raf complexes for activation of Raf-1 by protein kinase C. *Science* **280**: 109–111

Marcus KB, Bossoue SA and Patel AJ (1992) Myc function and regulation. *Ann Rev Biochem* **61**: 809–860

Marshall CJ (1995) Specificity of receptor tyrosine kinase signaling: transient versus sustained extracellular signal-regulated kinase activation. *Cell* **80**: 179–185

Oka H, Chatani Y, Hoshino R, Ogawa O, Kakehi Y, Terachi T, Okada Y, Kawaichi M, Kohno M and Yoshida O (1995) Constitutive activation of mitogen-activated protein (MAP) kinase in human renal cell carcinoma. *Cancer Res* **55**: 4182–4187

Owen AJ, Pantazis P and Antoniades HN (1984) Simian sarcoma virus-transformed cells secrete a mitogen identical to platelet derived growth factor. *Science* **225**: 54–56

Pumiglia KM and Decker JD (1997) Cell cycle arrest mediated by the MEK/mitogen-activated protein kinase pathway. *Proc Natl Acad Sci USA* **94**: 448–452

Sewing A, Wiseman B, Lloyd AC and Land H (1997) High-intensity Raf signaling cause cell cycle arrest mediated by p21Cip. *Mol Cell Biol* **17**: 5588–5597

Simon B, Weinel R, Hohne M, Watz J, Schmidt J, Kortner G and Arnold R (1994) Frequent alterations of the tumor suppressor genes p53 and DCC in human pancreatic carcinoma. *Gastroenterology* **106**: 1645–1651

Sivaraman VS, Wang H-Y, Nuovo GJ and Malbin CC (1997) Hyperexpression of mitogen-activated protein kinase in human breast cancer. *J Clin Invest* **99**: 1478–1483

Slamon DJ, Godolphin W, Jones LA, Wong SG, Keith DE, Levin WJ, Stuart SG, Udove J, Ullrich A and Press MF (1989) Studies of the HER-2 *neu* proto-oncogene in human breast and ovarian cancer. *Science* **244**: 707–712

Spandidos DA and Kerr IB (1984) Elevated expression of the human *ras* oncogene family in premalignant and malignant tumours of the colorectum. *Br J Cancer* **49**: 681–688

Stevenson MA, Pollock SS, Coleman CN and Calderwood SK (1994) X-irradiation, phorbolester, and $H_2O_2$ stimulate mitogen-activated protein kinase activity in HIH-3T3 cells through the formation of reactive oxygen intermediates. *Cancer Res* **54**: 12–15

Towatari M, Iida H, Tanimoto M, Iwata H, Hamaguchi M and Saito H (1997) Constitutive activation of mitogen-activated protein kinase pathway in acute leukemia cells. *Leukemia* **11**: 479–484

Vogel US, Dixon RAF, Schaber MD, Diel RE, Marshall EM, Scolnick EM, Sigal IS and Gibbs JB (1988) Cloning of bovine GAP and its interaction with oncogenic Ras. *Nature* **335**: 90–93

Vogelstein B, Fearon ER, Hamilton SR, Kern SE, Preisninger AC, Leppert M, Nakamura Y, White R, Smits AMM and Bos JL (1988) Genetic alterations during colorectal tumor development. *N Engl J Med* **319**: 525–532

Woods D, Parry D, Cherwinski H, Bosch E, Lees E and McMahon M (1997) Raf-induced proliferation or cell cycle arrest is determined by the level of Raf activity with arrest is determined by the level of Raf activity with arrest mediated by p21Cip1. *Mol Cell Biol* **17**: 5598–5621

Yao B, Zhang Y, Dellkat S, Mathias S, Basu S and Kolesnick R (1995) Phosphorylation of Raf by ceramide-activated protein kinase. *Nature* **378**: 307–310

Zheng CF and Kuan KL (1993) Cloning and characterization of two distinct human extracellular signal-regulated kinase activator kinases, MEK1 and MEK2. *J Biol Chem* **268**: 11435–11439

© 1999 Cancer Research Campaign 
